# Children-Robot Friendship, Moral Agency, and Aristotelian Virtue Development

**DOI:** 10.3389/frobt.2022.818489

**Published:** 2022-08-03

**Authors:** Mihaela Constantinescu, Radu Uszkai, Constantin Vică, Cristina Voinea

**Affiliations:** ^1^ CCEA, Faculty of Philosophy, University of Bucharest, Bucharest, Romania; ^2^ Department of Philosophy and Social Sciences, Bucharest University of Economic Studies, Bucharest, Romania

**Keywords:** imaginary companions, virtue ethics, Aristotle, social robots, children-robot friendship, human-robot friendship, virtue, moral agency

## Abstract

Social robots are increasingly developed for the companionship of children. In this article we explore the moral implications of children-robot friendships using the Aristotelian framework of virtue ethics. We adopt a moderate position and argue that, although robots cannot be virtue friends, they can nonetheless enable children to exercise ethical and intellectual virtues. The Aristotelian requirements for true friendship apply only partly to children: unlike adults, children relate to friendship as an educational play of exploration, which is constitutive of the way they acquire and develop virtues. We highlight that there is a relevant difference between the way we evaluate adult-robot friendship compared to children-robot friendship, which is rooted in the difference in moral agency and moral responsibility that generate the asymmetries in the moral status ascribed to adults versus children. We look into the role played by imaginary companions (IC) and personified objects (PO) in children’s moral development and claim that robots, understood as Personified Robotic Objects (PROs), play a similar role with such fictional entities, enabling children to exercise affection, moral imagination and reasoning, thus contributing to their development as virtuous adults. Nonetheless, we argue that adequate use of robots for children’s moral development is conditioned by several requirements related to design, technology and moral responsibility.

## Introduction

Advances in modern robotics have increased artificial agents’ sensing and visual processing capacities as well as mobility, pushing robots from industrial warehouses and factories to unlikely new places, such as hospitals, therapists’ offices, classrooms, and even homes (Darling, 2021). One of the most exciting avenues in robotics is the development of social robots relying on deep-learning artificial intelligence that can take on tasks that were traditionally assigned to human beings. For example, in recent years, there has been an increased interest in the design of social robots for the companionship and care of children (Dawe et al., 2019), with examples such as Embodied’s “Moxie,” SoftBank Robotics’ “Pepper,” and Blue Frog Robotics’ “Buddy.” Robots are intended to assist and even replace teachers, nannies, friends and children’s therapists (Sharkey and Sharkey, 2010; Zhang et al., 2019; Di Dio et al., 2020; Pashevich, 2021).

In this article we explore the moral implications of children-robot friendships using an Aristotelian framework. We argue that, although robot companions cannot be virtue friends, they can nonetheless enable children to exercise ethical and intellectual virtues, and thus contribute to children’s moral development. The reason is that the Aristotelian requirements for true friendship apply only partly to children: unlike adults, children relate to friendship as an educational play of exploration, which is constitutive of the way they acquire and develop virtues. We highlight that there is a relevant difference between the way we evaluate adult-robot friendship compared to children-robot friendship, which is rooted in the difference in moral agency and moral responsibility that generate the asymmetries in the moral status ascribed to adults versus children.

To develop this point, we look into the role played by imaginary companions (IC) and personified objects (PO) in children’s moral development and claim that robots play similar parts in children’s lives as these fictional entities. In particular, Personified Robotic Objects (PROs) can be important assets in enabling children to exercise affection, moral imagination, and reasoning, contributing to their development as virtuous adults. However, this contribution to children’s moral development is conditioned by meeting several requirements for adequate use, such as 1) responsible design that avoids positioning the robot companion as the equivalent of a human companion, for instance by avoiding situations when the robot reciprocates affection as if it were a true friend; 2) technically embedding scientific features that add new interesting challenges to kids’ reasoning abilities and make use of DIY toolkits with participative co-design; 3) proper ascriptions of moral responsibility to those who may bear blame for improper interaction between children and robot companions, such as parents or tutors, developers, marketers, etc.

## Children and Robots: Partners in Virtue or in Vice?

In this section we look into possible moral benefits and harms that interaction with robots might bring to children, in particular when this type of interaction is viewed as a form of companionship or friendship. We start with results from practice, which suggest that robots are accepted by children as they develop relationships based on trust and a common ground. Despite the potential benefits in children-robot interactions that empirical studies point towards, ethical problems still abound. We continue with theoretical accounts raising concerns, among others, over the potentially deceiving nature of robots engaging in relationships with humans.

### Children-Robot Interaction in Practice

Social robots are more than toys. Unlike usual toys, robots can move, interact, and communicate with children in complex ways. They can adjust their behaviour and responses to accommodate children’s specific needs and interests. In a world of over-crowded classrooms and overly busy parents, social robots that can look after children and provide companionship are indeed something to look forward to. This is why there is a growing interest in how children interact with robots in practice, as shown by the flurry of empirical research on this topic.

In an important study, Kahn et al. (2012) found that children attribute feelings, intelligence and interests (mental states) to the robot used in the study (Robovie) and they even see it as a potential partner or even friend. Indeed, the more similar the robot is to a human being, the easier it is for children to attribute mental states to the artificial agent, which means that robots can easily be considered part of the friends’ circle (Beran and Ramirez-Serrano, 2010; Manzi et al., 2020). Explaining to children that robots are “mere mechanisms” and not alive, does not seem to have a profound impact on how children attribute mental states to robots (Bartlett et al., 2004). But this does not mean that children are easily fooled by robots into considering them moral agents. Interestingly enough, children are not very eager to grant robots liberties or rights (Breazeal et al., 2016), despite attributing mental states to them. All of these studies seem to suggest that children see robots as semi- “moral others” (Nomura et al., 2016) which presupposes, some claimed, the emergence of a new ontological category (Kahn et al., 2004; Kahn et al., 2011; Kahn et al., 2012), as robots are seen by children as “neither alive nor not alive, but something altogether different.” (Severson and Carlson, 2010).

If indeed children perceive robots as potential partners, then it is no wonder that robots enhance learning processes (Kanda et al., 2004; Verner et al., 2016). Autonomous teacher-robots are considered credible by children, and they are successful in producing behavioural learning (intent to enact the recommendations made by the robot) (Edwards et al., 2016). Children can learn from robots especially when they can attribute to robots the ability to think and make decisions (Brink and Wellman, 2020). For example, robots can stimulate children’s curiosity and creativity (Ali et al., 2019a; Gordon, Breazeal, Engel, 2015). A scoping review confirms the potential benefits of social robots for learning, but also stresses the challenges–both technological and pedagogical–that must be addressed before introducing artificial agents into every classroom (Belpaeme et al., 2018). All in all, robot teachers are more effective than computer-based tutoring, probably due to the embodied nature of the experience (Belpaeme et al., 2013).

Another field where robots seem to be effective is healthcare. Robot Assisted Therapy is a promising field in paediatrics, as robots have successfully been used to provide companionship and alleviate loneliness for children during therapy and hospital stays (Blanson-Henkemans et al., 2012; Belpaeme et al., 2013). But nowhere have results been more promising than in robot therapy for autism spectrum disorders (ASD) (Coeckelbergh et al., 2016). One study shows that children with ASD interacted more with a social robot than with a human interlocutor—a fact that can be partly attributed to children’s excitement and curiosity regarding the robot (Kim et al., 2013). Yet other studies found that social robots facilitate the learning of some social rules in children with ASD (Ali et al., 2019b; Zhang et al., 2019). Another review points towards the fact that robots do indeed elicit more engagement and also novel social behaviours especially from children and teenagers with ASD (Scassellati, 2007; Scassellati et al., 2018). Researchers do not know why children with ASD are more responsive towards robots, but they speculate that amongst the reasons might be the fact that interaction with robots eliminates the stress of social interaction present in communication with other human beings. In other words, interaction with robots means less social overstimulation for children, as robots offer more predictable and reliable responses than humans do (Scassellati, 2007; Darling, 2021).

There is nonetheless a lack of longitudinal studies on how robots influence children’s social and emotional development. One reason is that today’s robots created especially for children’s care are not made for interactions that are longer than a couple of weeks or months; it is thus difficult and maybe too early for researchers to track children-robot interaction over the years (Pashevich, 2021). Another issue that complicates the study of children-robot interaction is that advanced robotic technologies are still very new, expensive, limited and not robust enough to be used in day-to-day life for long periods of time, such as years. Likewise, large-scale empirical studies involving such new and emerging technologies might face a barrage of objections from research ethics committees. Moreover, many studies mention the “novelty effect” of interacting with robots, which basically boils down to the fact that most children enrolled in the studies are at their first encounter with complex robots and this might explain their mostly positive reactions to artificial agents (Michaelis and Mutlu, 2018). Furthermore, robotic technologies are very diverse, both in their functions, as well as in their appearance. The context (place and culture) in which the interaction takes place, as well as the intervention the robots are used for (teaching, therapy, nursing etc.) might all have a say in shaping children-robot interactions. This is why even if empirical results are promising, they are not enough, by themselves, to dispel the theoretical concerns sketched below.

### It is All Fun and Games Until Someone Ends Up Vicious

More and more worries regarding the potential pitfalls of human-robot interaction have been raised in the last few years. Among them, taking robot companions to be friends has been discussed as negatively affecting human users’ moral, emotional, and social abilities, because: 1) considering the robot to be a genuine friend is deceptive (Elder, 2015; Nyholm 2020; Sharkey and Sharkey 2021), 2) undermines the value of social connections (Jecker, 2020; Prescott and Robillard, 2021; Sharkey and Sharkey, 2021), 3) does not contribute to the development of virtue, but only of vice (Nomura et al., 2016; Sparrow, 2016), and 4) leads to moral deskilling (Vallor 2016). In this section we focus on these concerns and highlight especially the dangers that introducing social robots into children’s lives might bring about for children’s moral development.

The most pressing issues in adult-and children-robot interactions have to do with the deceiving nature of the relationship (Sparrow and Sparrow, 2006; Elder, 2015; Danaher 2020; Sharkey and Sharkey, 2021). As long as robots only simulate the reciprocation of friendship, treating them as friends would only amount to a simulacrum of friendship (Nyholm, 2020). In other words, social robots only feign human socio-emotional reactions and behaviours, thus any goodwill or affection towards them would be one-sided (Matthias, 2015), leading to self-deception (Navon, 2021). According to Sharkey and Sharkey, deception arises whenever “the appearance and the way that a robot is programmed to behave, creates, for example, the illusion that a robot is sentient, emotional, and caring or that it understands you or loves you” (2021, 311). There are still ongoing debates on what deception is and under what circumstances it arises, for example, whether it requires intention or not (Sparrow, 2002, 2016; Elder 2015; Matthias, 2015; Danaher, 2020), but under all views, deception is morally problematic in view of its effects on the deceived or on society at large (Sharkey and Sharkey, 2021).

In the case of children, who are an epistemically vulnerable group, deception could further result in undermining the value of social connections (Jecker, 2020; Prescott and Robillard, 2021; Sharkey and Sharkey, 2021). Since growing up with artificial companionship would be easier and less challenging than trying to develop a friendship with a human peer, one might argue, along the lines of Turkle (2017) that constant interactions with robots might disincentivize children (and, as a result, future adults) from pursuing relationships with other humans. In a similar vein, (Sharkey and Sharkey 2010; Sharkey and Sharkey 2021), drawing upon research from developmental studies on attachment, argue that robot companionship (especially in the early stages of childhood) could end up harming children by not equipping them properly for socialising with other humans. Parents could also be “fooled” into believing that the robots meet the emotional demands of children. This, in its turn, could lead to robots being allocated social roles for which they are unfit. Just like some schools might make a push for having robot teachers in a time poised with staff shortages (Sharkey, 2016; Sharkey and Sharkey, 2021) parents might actively “push” children into treating their robot as a friend. Such a relationship has the potential to damage children both physically (a robot friend might not be able to adequately distinguish all the time between a child’s adequate use of scissors) or emotionally (Sharkey, 2016; Sharkey and Sharkey, 2021). The deception argument in the case of children emphasises the fact that virtue cannot be developed and exercised outside the scope of society, but only when interacting with other human agents around them. Then, with less social connections with human beings and more connections with social robots that apparently satisfy children’s social and emotional needs, kids might end up having less opportunities to develop their moral character.

An additional worry regarding true friendship with robots relates to the fact that human-robot interaction might not contribute to the development of virtue, but only of vice in the case of children. On the one hand, take, for example, Sparrow’s intriguing cases of kicking robot dogs (2016). Kicking a robot dog is morally problematic not because of the impact that such an action might have on the object, but because doing it reveals something about our dispositions: we have a cruel and irascible moral character. In other words, the way in which we interact with robots gives us a chance to show whether we have a vicious moral character. But because robots do not normally “object” or respond to abusive behaviour, children might be incentivized to experiment cruel behaviour on them. This might desensitise or habituate children with vices, which is especially worrisome if, as Nomura et al. (2016) showed, children might derive pleasure from abusing their robot companions.

On the other hand, if people act virtuously towards robots, this will not prove that those people are virtuous agents. This is due to the fact that robots are deprived of any moral status; thus, there is no one there to take advantage of that kindness. The crux of this asymmetry rests upon the role which practical wisdom plays in vicious or virtuous behaviour. While vicious behaviour does not need to be guided by practical wisdom, virtuous behaviour does. It is for this reason that, according to Sparrow (2021, 5), “a person who possessed practical wisdom would know that and would also therefore realise that “kindness” towards robots does not realise the goals—the improvement of the welfare of humans and (perhaps) other sentient creatures—towards which kindness is oriented. In the absence of the exercise of practical wisdom, virtue is impossible and what would ordinarily count as the exercise of a virtue—the demonstration of a kind disposition—fails to do so.”

Another possible detrimental effect of taking robot companions to be genuine friends relates to children’s moral deskilling. This worry has been taken up by Shannon Vallor, (2015); Vallor, (2016), who claims that robot companions could ‘morally deskill’ and thus prove to be “morally debilitating” for people in their capacity to develop virtue friendship with other human beings. According to Vallor, our moral skills (and this is a point of utmost importance to children, as prospective moral agents) are “typically acquired in specific practices which, under the right conditions and with sufficient opportunity for repetition, foster the cultivation of practical wisdom and moral habituation that jointly constitute genuine virtue” (2015, 109). As a result, in order to develop moral skill and cultivate phronesis, a child needs to be exposed to 1) models of skilful practice, 2) the proper institutional context (namely the cultural and social norms) which reinforce such a skilful moral practice, 3) possess proper basic moral motivation, 4) possess the necessary cognitive and emotional resources, and 5) and environment with a constant feedback loop that gives us the opportunities to habituate and learn from failure (2015, 110).

While the possession of conditions 3) and 4) might simply rest on the general moral and cognitive upbringing of the child, for the remainder of them acquisition becomes more problematic in the context of robot friendships. Take, for example, the role that the exercise of patience (Turkle, 2017; Nyholm and Frank, 2017) has in fostering genuine relationships and virtue friendship with other human beings. While children will search for instant gratification in relation to robot companions, the latter would need to possess practical wisdom and a moral personality to understand why patience is important in developing a relationship with another human being and how that is fostered by our social and cultural institutional context.

Most of the above theoretical concerns share an Aristotelian understanding of moral development and virtuous life. In this framework, real friendship is an important element in the acquisition and development of virtue. Given that robots cannot at the moment but simulate human mental and emotional capacities, most researchers conclude that friendship with robots can only be an illusion (Sparrow and Sparrow, 2006; Elder, 2015; Turkle, 2017; Sharkey and Sharkey, 2021).

In what follows, drawing on the Aristotelian conception of the value and role of friendship and the broader framework of virtue ethics, we build the case that robot companions might nonetheless have a positive impact on the moral development of children, given the difference between the way we evaluate adult-robot friendship compared to children-robot friendship.

## Robot Friendship, Moral Agency, and Virtue Ethics

Most of the current accounts concerned with friendship between humans and robots are grounded in the Aristotelian account of friendship and virtue (Elder, 2015; Elder, 2017; de Graaf, 2016; Danaher, 2019; Nyholm, 2020) or at least take it into consideration (Ryland, 2021). As a result, the focus of contemporary debates is on delineating the criteria for what Aristotle calls “virtue friendship” and see whether human-robot friendship satisfies them. With but a few notable exceptions (Danaher, 2019), most researchers argue that current and foreseeable robots are unable to be our virtue friends (Elder, 2015; de Graaf, 2016; Nyholm, 2020). However, adopting different theoretical perspectives allows some to confer human-robot friendship a relevant moral role (Gunkel, 2018; Marti, 2010; Ryland, 2021) and reject the standard Aristotelian account as being too demanding. Yet others highlight that such alternative contemporary accounts endorse “some fairly watered-down and less interesting sense of friendship” (Nyholm, 2020: Sect. 5.7).

In this article we follow the mainstream approach and adopt the standard Aristotelian view of friendship. The main reason for taking this stance is that we consider it is still robust and relevant today, in particular because the broader framework of virtue ethics has already proved useful in inquiries over moral status, moral agency and moral responsibility in the fields of machine ethics (Wallach and Allen, 2008; Howard and Muntean, 2017) and Human-Robot Interaction (Cappuccio, Peeters and McDonald, 2020; Peeters and Haselager, 2021), and even more when applied to robotic AI systems (Hakli and Mäkelä, 2019; Coeckelbergh, 2020; Sison and Redín, 2021; Constantinescu et al., 2022). Another reason is because we consider that alternative contemporary accounts of friendship did not (yet) provide sufficient grounds for us to give up the standard Aristotelian account, but rather to annotate it.

We discuss below the relevance of moral agency and moral status for human-robot friendship by drawing on Aristotelian virtue ethics and contemporary criteria for friendship rooted in the standard Aristotelian view. Our main claim is that Aristotelian criteria are robust enough for inquiries over adult-robot friendship but have some limitations when applied to children-robot friendship. These limitations can be explained through the differences in virtue, moral agency, and moral responsibility of children, compared to adults. We find the asymmetry in moral responsibility and moral agency between children and adults, and between children and robots to be of particular interest when evaluating the possible relation of friendship between children and robots.

### Aristotelian Criteria for True Friendship and Characteristics of Good Friends

As laid down in Aristotle’s *Nicomachean Ethics* ^*1*^ (books VIII-IX), friendship (Gr. <*philia*>) is of three species, corresponding to the three reasons that make someone worthy of love (VIII, 3): 1) for utility, in view of gaining some personal benefit, 2) for pleasure, as the parties are guided by feelings in enjoying each other’s pleasant presence, and 3) for virtue or for good (character), between “those who are alike in their virtue: they each alike wish good things to each other in so far as they are good, and they are good in themselves” (VIII, 3, II56b5-10). While the first two species of friendship are imperfect because they are instrumental, the third one is considered by Aristotle to be true or complete friendship because it is pursued for itself.

Contemporary research concerned with human-robot friendship (Danaher 2019; Elder, 2015, Elder, 2017; de Graaf, 2016; Nyholm 2020; Ryland, 2021) have picked up this Aristotelian view of friendship and proposed various sets of criteria for perfect or virtue friendship (see Danaher 2019 and Ryland, 2021 for classifications of main conditions referenced in the literature). These conditions include, for instance, mutuality, authenticity, equality, empathy, shared life, or associative duties. While we consider the various conditions put forward to be relevant and well-grounded in the Aristotelian account of friendship, we think that they tend to ignore or place too little emphasis on the characteristics of good friends as depicted by Aristotle. This has a detrimental effect on the way we engage with evaluations of human-robot friendship, leading to failure to acknowledge the relevance of virtue, moral agency, and moral responsibility for friendship within the broader account of Aristotelian virtue ethics.

In Aristotelian virtue ethics, the right or virtuous action in a particular context is the action that a person who acquired virtues would choose to do: “A virtuous action in certain circumstances is what is required in those circumstances and what a virtuous person would do in those, or relevantly similar, circumstances” (Crisp, 2015, p. 270). A right or virtuous action can thus only result from a virtuous character (NE, 1106b18-24), as “there is no such thing as an objectively virtuous action in itself, considered independently of the person who performs it” (Sison and Ferrero, 2015: 86). It takes practice and habituation to acquire and develop the necessary stable and enduring states or dispositions (*hexeis*) of character that enable agents to correctly grasp the right thing to do in specific circumstances, that is, ethical virtues (*ethike areté*). Furthermore, ethical virtues are developed through the exercise of reason and are made possible through the enabling role of intellectual or dianoetic virtues (*dianoetike areté*), which include knowledge of the necessary and eternal (the virtues of *episteme, nous*, and *sophia*) and knowledge of the contingent and contextual (the virtues of *techné* and *phronesis*) (Irwin 1999; Mureșan, 2007; Meyer 2011; Crisp 2018). Thus, ethical virtues rest on rational choice or deliberation (*prohairesis*) (NE 1106b36 and 1139a22), and are guided by practical wisdom (*phronesis*), which enables agents to rightly deliberate across various contexts of action (Irwin, 1999).

When we relate the broader claims of virtue ethics to characteristics of good friends, we find it important to highlight that to be a friend in the virtue friendship sense one needs to be good, that is, virtuous (Nyholm, 2020). This happens because virtue friendship takes place between good or virtuous people, who lead a virtuous life. It is precisely because of their being good or virtuous that such people are able to commit to virtue friendship (NE VIII, 4, II57a30). They show friendship towards each other “because of what they are, not for any other incidental reason” (NE VIII, 3, II56b, 10–15). Interestingly, Aristotle notes that friends involved in virtue friendship are like alter-egos, as a good friend is another self of the good person (NE IX, 89, II69b5): virtuous people become friends and love one another for their characters (Crisp, 2018). As Aristotle notes, friendship consists more in loving than being loved, rendering loving the virtue of friends (VIII, 8, II59a35). For the good person acts towards the good or the virtuous, having in mind the sake of their friend, even if this means neglecting their own interest (IX, 7, II68a35). As a result, empathy is an important condition for virtue friendship (II66b34), understood as caring for the good of the other, grieving and joying along with them. It is through empathy that the friend becomes another self.

Furthermore, the process of developing or training virtues takes place when good people live in each other’s company (IX, 9, II70a10), because they become better and improve each other through their activity (IX, 12, II72a10). True friendship requires familiarity and time (VIII, 3, II56b, 25), experiencing interaction in various situations. This is why friendship is both a virtue and involves virtue (Crisp, 2018). This further explains why friendship is also mutual and rests on rational choice: friendship is a state rather than a feeling, a state that “makes people wish good things to those they love, for their sake” (VIII, 5, II57b30). One important implication of the Aristotelian framework is that true friendship is between equals (VIII, 5, II58a). There can be, however, particular species of genuine friendship involving superiority, such as that between father and son or older to younger (VIII, 7, II58b10). This means that the type of equality required between parties engaged in virtue friendship involves equality in moral status (Nyholm, 2020).

At this point we seem to run into an obvious obstacle for human-robot friendship: despite some openness to grant robots moral standing (Gunkel 2018), most researchers would agree that there is a difference in the moral status ascribed to robots as opposed to human beings. Currently, the standard (and only) entities that are ascribed full moral agency and moral responsibility, which are the building blocks of what we call moral status, are adult human beings (Hakli and Mäkelä, 2019). Moreover, researchers highlight the incapacity of robotic AI systems to be virtuous and thus take part in virtue friendships (Nyholm, 2020; Constantinescu and Crisp, 2022). The discussion over human-robot virtue friendship seems to ultimately revolve around the moral status that we ascribe to robots and, relatedly, to the possibility of them being moral agents. Similar to other researchers adopting the standard Aristotelian view, we hold that human-robot friendship cannot be considered virtue friendship. However, in reaching this conclusion we do not weight human-robot friendship against a specific set of Aristotelian criteria, but instead highlight that robots are unfit to be virtue friends because they lack the required moral agency to make them part of our moral community, that is, agents capable of virtuous or vicious action for which they can receive blame or praise.

But what happens when neither robots, nor humans engaged in human-robot interaction are full moral agents? Is it possible to speak of virtue friendship in this case, given the faded difference in moral status? We continue with a few remarks over the implications of moral status and moral agency in the case of children-robot robot friendship, as opposed to adult-robot friendship.

### Robot Friends, Moral Development, and Moral Agency: Childhood Versus Adulthood

Building on Aristotelian virtue ethics, we would like to highlight that there is a difference between the way we evaluate adult-robot friendship compared to children-robot friendship. This is rooted in the difference in moral agency and moral responsibility that generate the asymmetries in moral status ascribed to adults versus children.

Contemporary research discusses two main conditions to ascribe moral agency and moral responsibility to an entity, which are grounded in Aristotelian virtue ethics ( Coeckelbergh, 2020; Constantinescu et al., 2021; Sison and Redín, 2021): the freedom and epistemic conditions. These conditions pick on Aristotle’s discussion over criteria to ascribe blame or praise to an agent depending on their acting viciously or virtuously, introduced in his *Nicomachean Ethics* (Book III parts 1 to 5 and Book V parts 8 and 9). In the Aristotelian framework, conditions for moral agency and moral responsibility are inherently intertwined with conditions for virtue of character, for it is only when individuals act as a result of their virtue or vice that we might hold them praise- or blame-worthy (Meyer, 2011). Drawing on these Aristotelian distinctions, in order to be a moral agent and bear moral responsibility, one needs to be able to 1) causally generate an outcome while 2) acting freely, uncoerced, and 3) be knowledgeable of the contextual circumstances of their action, following 4) deliberation based on rational choice, involving reason and aforethought (for a broader discussion see Constantinescu et al., 2022). Aristotle’s discussion over these four criteria highlights that children are not yet moral agents because they lack deliberation (*prohairesis*), which is a constitutive condition of moral agency and moral responsibility, requiring agents to be able to act based on rational choice.

This difference in moral status generates important differences in the moral development of children engaging in friendly interactions with robots, as opposed to adults engaging in friendship with robots. On the one hand, when adults direct virtuous behaviour towards robot companions, they seem to fail to understand that robots are not appropriate targets for kindness, for instance (Sparrow, 2021). As a result, adults engaging in apparently true or virtue friendships with robot companions show that they lack the required practical wisdom that guides morally responsible agents. On the other hand, when children direct virtuous behaviour towards robot companions, we might rather regard this as a learning process towards becoming virtuous. Because children are not yet full moral agents, we do not (and should not) hold the same expectations of them when it comes to developing forms of friendship with robot companions. When children bond with robot companions and direct virtuous behaviour towards them, the reason is not because of failing to grasp the context and failing to exert practical wisdom. This rather happens because childhood is the age where individuals are trained to acquire and to develop virtues. In other words, children do not yet have the life experiences necessary for developing practical wisdom.

As a result, we consider that robot companions might have a positive impact on the moral development of children. Children-robot friendship is not and cannot be virtuous because both parties lack the moral agency required for a virtuous moral status normally ascribed to adult human beings, as laid down by the Aristotelian theory of moral responsibility. However, Aristotle himself notes that, as concerned with the young, friendship keeps them from making mistakes (VIII, 1, II55a15), despite the fact that the young only form friendships for utility and for pleasure, because they “live in accordance with their feelings” (VIII, 3, II56a30-35). Though not true or perfect friends, robot companions envisioned, along Aristotelian lines, as friends for pleasure or utility, might contribute to children’s ability to acquire and develop virtues, for example through the characteristic activity of childhood, that is, play. If robot friends contribute to children becoming virtuous, this could further have a positive impact on the way children exercise moral deliberation on their path toward becoming morally responsible adults. Nonetheless, this contribution to children’s moral development is conditioned by meeting several requirements for adequate use. We discuss these issues in the next section of our article.

## Personalised Robotc Objects, Friendship and Children’s Moral Development

Friendship has received a great deal of attention, but its paradigmatic example is adults’ relationship. Rarely has the issue of friendship between children and for children been in the spotlight, because “the general attitude tends to be a “bus theory” of friendship: do not worry if you miss one, another will be along in a minute” (Healy 2011, 442). This attitude towards children’s friendship has probably been informed by a misreading of Aristotle’s view on friendship and its importance for children. Indeed, children are not capable of virtue friendships because they do not really possess virtues as relatively stable traits of character (Kristjánsson, 2020). But, as we previously showed, while Aristotle mentions that children can only form friendships for utility and pleasure, he nonetheless acknowledges the formative dimension of these imperfect relationships for the development of children’s characters: “the young need [friendship] to keep them from error” (NE 1155a). Childhood is the time when individuals learn to become virtuous and develop as future moral agents. Friendship, even in imperfect kinds, such as for pleasure or utility, is constitutive of children’s moral development.

In this section we draw an analogy between social robots and imaginary companions (IC) and personalised objects (PO) to show that “imagined” and one-sided relationships of friendship are not uncommon for children. Children have always used their imagination to come up with new play friends, as is the case with IC and PO (such as stuffed toys). What is more, IC and PO help children learn about social relations, affection, care, and responsibility - which is what robot companions can do well. We position robot companions as Personalised Robotic Objects (PROs) and explore the way they might enable children to exercise primarily their intellectual virtues, and secondarily their ethical virtues, thus contributing to children’s moral development. We further develop several conditions that need to be met so that adequate use of PROs may indeed contribute to the exercise of virtues through childhood.

### New Kids on the Block Practice Their Virtues

Children have always had imaginary companions. Scientific research on imaginary companions (IC) evolved during the last decades from a very restrictive position–IC are a sign of possible mental disorders–towards a more inclusive, less medicalised one: ICs are common and normal, even beneficial to the psychological and social development of children. A longitudinal study from 2004, so before the advent of social media and smartphones, found out that 65% of 7-year-old kids had at least one imaginary friend at some point in their childhood, and 28% of 12-year-old children still have them (Taylor et al., 2004, 1182–83). ICs are for impersonation or “pretend play”; this “fantasy behaviour” is not detrimental, there is no evidence that having IC will produce negative personality traits (Taylor et al., 2004, 1182). It seems that in this aspect kids with IC are not different from those lacking a penchant for fantasy. Newer approaches to this topic, even when investigating the similarity between IC and hallucinations, recognize the fact that “creation of fictional characters and the generation of imaginary friends arguably share a feeling of distributed agency paired with knowledge of the subjective source of these creative acts” (Fernyhough et al., 2019, 8). Furthermore, children with IC tend to outclass the less imaginative ones in the domain of mental state reasoning and social competence, even though it is debatable whether having IC betters one off or IC is a result of one’s advanced abilities (Davis 2020, 380–81). Research shows that kids are very serious about their play. This is why children tend to treat imaginary companions as real-life, flesh-and-bone friends (Davis 2020, 382).

Furthermore, personified objects (PO–that is, tangible imaginary companions, such as stuffed teddy bears), as fantasy predispositions, are isomorphic with IC, as the activity involved is the same: creating another entity for the sole purpose of interacting with that presence, be it material or just mental (Davis 2020, 376). The difference between the two rests in the infinite possibilities of mental design of IC compared to the limited set of shapes and behaviours of PO, such as social robots or interactive toys. It is more challenging to design and attribute a mind to a complex still lifeless machine that already has a given form with its functions and affordances. Beyond this difference, both IC and PO serve the purpose of exercising children’s moral imagination which, in its turn, is constitutive in the process of developing the intellectual virtue of practical wisdom. Moral imagination is “the capacity to envision given contexts from multiple, even incompatible, frames of reference to ensure a broadened moral lens with which to approach and assess lived experience” (Fletcher, 2016). The exercise of moral imagination is especially important in childhood as it helps children take note of and distinguish the ethically relevant features in different contexts. What is more, moral imagination is necessary for imagining situations not yet encountered in experience and to compare them to alternative situations. In other words, moral imagination is a sort of “anticipatory moral appraisal” that helps people confronting moral dilemmas to imagine alternatives that are practically and ethically feasible and sound (Werhane, 1999).

Social robots could, in a sense, play a very similar role in children’s lives as imaginary companions and personified objects do. Children tend to imagine and project human-like characteristics to all sorts of objects, be they inanimate or just imaginary. The worry that we developed in the previous sections is that children might do the same with social robots. We claim that all the pretend activities that children engage in, such as imagining the character, emotions, mental states or even words of IC, PO or social robots are similar because they involve role play, more precisely, imagining what it is to be in another’s shoes.

Social robots differ from imaginary companions because they have an already set appearance, while ICs could take an almost infinite set of shapes and voices. Moreover, ICs and POs evolve and grow with children, meaning that their characters and features change as children develop both emotionally and cognitively. In other words, ICs grow and develop alongside their creators. But social robots are not entirely constructed by children, as they already come embedded with a predefined set of responses and actions (even if there is a possibility for social robots to adapt their responses to their users’ preferences). This might, in fact, reduce the chances of children becoming fascinated and absorbed by social robots, given that sooner or later the cognitive and emotional limitations of the robots will become apparent to children. What research on ICs and POs teaches us is that as long as robots are integrated into children’s play practices, they could very well serve as instruments that support the development of children’s ethical and dianoetic virtues, just as children’s imaginative practices of creating imaginary companions do.

Along these lines, we suggest that children-robot friendship, though not a true (virtue) friendship in the Aristotelian virtue framework, plays a similar role to children’s moral development compared to IC and PO and contributes to the development of ethical and intellectual virtues. In our assessment of children-robot friendship, we should therefore focus on the role that a social, relational robot can play. We understand the role of a robot companion or robot friend as a PRO–Personalised Robotic Object. We continue with discussing some requirements for adequate use of PROs as enablers for children’s moral development.

### Like a PRO! But Some Restrictions Apply

When children say they want robots to be their friends, this is actually an invitation: “Let’s play the friendship game.” This play is not just aimless entertainment, but an active method of using imagination, affection, and reason in order to recognize the perspectives of other (non-human) entities (Davis 2020, 374). Our claim is that children-robot friendship can play a positive role in children’s moral development, through the development of both ethical and intellectual virtues. Furthermore, we suggest that PROs would have a more direct impact on the acquisition and practice of intellectual virtues, and a rather indirect impact in what concerns ethical virtues. Nonetheless, we complement our optimism over the positive role that PROs might have for children’s moral development with precaution over requirements for sound deployment of PROs. We now turn to discussing PROs as enablers for children’s moral development, together with the conditions under which PROs might indeed help children develop as virtuous agents.

First, through their bonding with robot companions, children exercise the type of dispositions of the soul that enable future development of ethical virtues. Empirical studies in children-robot interaction presented in the section on “Children-robot interaction in practice” show that children consider robots their friends, they engage in play with these artificial companions, and even learn from them. In other words, children feel affection towards robots, although they regard robots as belonging to a special new ontological category. According to Aristotle, affection and subsequent care are two natural dispositions of the soul that must be developed to strengthen a good, virtuous character. Still, “affection for soulless objects is not called friendship, since the affection is not mutual” (*NE* II55b30) at most, it can be called goodwill (*eunoia*). Goodwill is a necessary but not sufficient condition for true friendship, as it is complemented, among others, by reciprocity. Nonetheless, allowing children to form affection bonds with robots, although presupposes a non-mutual type of relationship, can contribute to habituating children with exercising goodwill and responsibility towards others. This, in its turn, can indirectly support the development of ethical virtues in children.

On the cautionary side, we would like to emphasise that it is important that children (and adults taking care of them) understand that the type of friendship established with PROs is not the equivalent of true friendship and that it may not replace friendship with humans—which means that we need to think of PROs foremost from the perspective of responsible design. On the one hand, children should not mistake robot companions for human companions, and one way to avoid this situation is to avoid designing robots that “express social cues to deliberately facilitate bonding with them” (Bartneck et al., 2020, 195). The seemingly reciprocal affection that robots would display is one based on modelled calculations and not genuine feelings, which are constitutive of true friendships. Designing robot companions to behave as if they were true friends, able to reciprocate virtue-based affection is misleading, because it gives the impression that the technology is more advanced than it really is (Riek, 2012). Similar to the case of IC and PO, it is important to keep explicit the lack of moral agency in the case of robots, while allowing for one sided emotional engagement (Bryson, 2018; Mamak, 2021). On the other hand, the way children interact with robots would need to mimic their interaction with humans for it to be a good, relevant exercise for their adult virtuous development: “What is important is that children interact with robots in certain ways, and conceive of them in certain ways, that are similar in some respects to how they interact with and conceive of humans” (Boulicault et al., 2021, 7). Therefore, to fit relevant concerns raised by many in relation to robots designed with human appearance and functions (Bryson, 2010; Bryson, 2018; Coeckelbergh, 2016), one potential requirement for PRO design is that the robot-companion could position itself as a robot in dialogue with the child user, stating what it can and cannot do, basically stating the limits of the game of child-robot friendship.

Second, relationships with robot companions may contribute to the development of dianoetic or intellectual virtues in children, which are an important acquisition in themselves and are also essential for the moral growth of a good character. The scientific and technical nature of robots could add new interesting challenges to kids’ intelligence and contribute to their reasoning abilities. PROs could thus provide a good stimulus for studying, experimenting, discovering, tinkering, hacking, for instance. In particular, PROs may enable knowledge for what is necessary and what is contingent or of what is invariable and what is variable (Crisp, 2018, xxiv), contributing to the development of the intellectual virtue of practical wisdom, which can later guide them to interpret lived experiences as adults.

An important requirement here is that due attention must be paid to technical features underlying robot interaction with children. One condition for building PROs in a responsible way might involve co-designing them with children (and adults taking care of them) (Boulicault et al., 2021), hence, to value their participation and input. Participative co-design has already proved useful for therapy (see Aslam, van Dijk and Dertien, 2019) and is consistent with the Aristotelian ethical framework, enabling the exercise of intellectual virtues such as scientific knowledge or skill knowledge, as well as of ethical virtues such as perseverance and patience. For PROs to have such a positive impact on children development, one related requirement might regard designing the robot-companions as open-source toolkits that encourage children to experiment, tinker, and imagine, among others. Available examples include DIT friendly toolkits described by Aslam et al. (2019) or Vandevelde et al. (2017).

Third, because social robots offer more predictability and less unclear signals than other humans do, and because children try to adapt their preferences to the available options (Elder, 2017, 123–24), interpreting robots as friends becomes an exercise in socialisation and imagination, thus an exercise of their capacities of seeing certain contexts and relations from multiple points of view. For children, friendship and play are valuable and go hand in hand. (Elder, 2017, 121) properly observed that “playful simulacra can be important in helping children learn skills that will help them to better navigate the real thing.” The key is in the relationship’s *making*, which happens within the specific activity of play. To see robots as “artificial” friends is hardly any different than developing friendships with an imaginary companion or a personified object: it is a “moral laboratory.”

Related concerns here regard deception and substitution in children-robot friendship. Nonetheless, worries regarding the deceptive nature of children-robot interaction, while warranted in principle, should not be overplayed. If the wrongness of deception in robotics is not inherent, but contingent upon the way in which we assess the implications of children having such interactions, then in some cases potential deception might not only be “harmless fun” (Sharkey and Sharkey, 2021, 311), but even beneficial playfulness (Elder, 2017). Furthermore, while we acknowledge that due consideration needs to be given to the potential substitution effect (Sparrow and Sparrow, 2006; Sharkey and Sharkey 2010), we would like to point out that playing the friendship game with PROs might complement and enhance human friendships (Danaher, 2019). As Ryland (2021: 391) puts it: “it is arguable that befriending robots (to some degree) could actually improve the extent to which we tolerate and include other human beings.” A suggestion to conceive and design PROs with due precaution for deception and potential substitution effect is therefore to direct the children-robot friendship towards interaction that enables virtue development on children’s side. This means taking into account the lack of moral agency and responsibility in both robot-friends and child-users and envisioning the role of robots as supporting virtue development in children’s lives as a whole, beyond their interaction with robots.

Finally, due precaution needs to be paid to possible harms resulting from children interacting with PROs and ascribing proper responsibility and blame. There is yet no definite answer regarding the network of moral responsibility surrounding robot deployment and it is not within the scope of the current article to offer one. Nonetheless, possible candidates for children-robot friendship resulting in harmful moral outcomes include at least parents or tutors, designers, programmers, manufacturers, and marketers. What is important to highlight is that, given the special nature of the two parties involved in children-robot friendship, with none being a moral agent, blame, and responsibility for each party is to be borne by someone else, and this also covers aspects related to the amount of time that children spend with their PROs, the functions that are activated on the PRO relative to children’s group age, or the way the PRO is empowered to occupy a certain role in the household, to name but a few.

## Conclusion

What practical lessons can be derived from this perspective on the role of PROs in children’s moral development? First, designers should consider the vastly different types of relationships children can have, not only with other humans, but also with imaginary companions and personified objects. Unlike adults, children are not yet constrained by social norms, thus they have a much bigger space in which play serves the purpose of discovering, learning, and imagining new situations and life-contexts. PROs should thus be co-designed alongside children in order to stimulate moral imagination and the development of ethical and dianoetic virtues. Second, PROs should not be marketed for a specific role or as a substitute to friends, teachers, pets, and so on. The task of imagining the roles PROs should play in children’s lives should be left to the beneficiaries of these technologies, that is children and their tutors. Last but not least, special attention should be paid to issues related to safety, reliability and trustworthiness, which we have not addressed in this article, given our focus on the implications of children-robot friendship for virtue development. For instance, the use of robot companions for children raises important questions of privacy and surveillance, which is an issue for AI embedded technologies in general. Robots should not become additional tools of surveillance and control, and this is an issue that should not be left to be solved by industry self-regulation. Additional concerns regarding moral and legal responsibility thus arise when discussing children-robot friendship.

Still, many things remain to be discussed, such as the place to be ascribed to robots in the established relationship between children and their parents, teachers, or friends. A co-design paradigm is undoubtedly recognizing children’s input and perspectives, but once the PROs come out the factory gate, should children be allowed to tinker their own robots, to mod them like a game console, to modify them to suit their own interests beyond the producers’ bounds?

## Data Availability

The original contributions presented in the study are included in the article/supplementary material, further inquiries can be directed to the corresponding author.
